# Imatinib mesylate (Gleevec) downregulates telomerase activity and inhibits proliferation in telomerase-expressing cell lines

**DOI:** 10.1038/sj.bjc.6602592

**Published:** 2005-05-03

**Authors:** O Uziel, E Fenig, J Nordenberg, E Beery, H Reshef, J Sandbank, M Birenbaum, M Bakhanashvili, R Yerushalmi, D Luria, M Lahav

**Affiliations:** 1 Felsenstein Medical Research Center, Beilinson Campus, Sackler School of Medicine, Tel Aviv University, Petah-Tikva, Israel; 2 Institute of Oncology, Beilinson Campus, Sackler School of Medicine, Tel Aviv University, Petah-Tikva, Israel; 3 Institute of Pathology, Assaf Harofeh Medical Center, Zerifin, Israel; 4 Division of Infectious Diseases, Sheba Medical Center, Tel-Hashomer, Israel; 5 Medicine A, Rabin Medical Center, Beilinson Campus, Sackler School of Medicine, Tel Aviv University, Petah-Tikva, Israel

**Keywords:** telomerase, imatinib mesylate, proliferation

## Abstract

Imatinib mesylate (IM) is a tyrosine kinase inhibitor, which inhibits phosphorylation of downstream proteins involved in BCR-ABL signal transduction. It has proved beneficial in treating patients with chronic myeloid leukaemia (CML). In addition, IM demonstrates activity against malignant cells expressing c-kit and platelet-derived growth factor receptor (PDGF-R). The activity of IM in the blastic crisis of CML and against various myeloma cell lines suggests that this drug may also target other cellular components. In the light of the important role of telomerase in malignant transformation, we evaluated the effect of IM on telomerase activity (TA) and regulation in various malignant cell lines. Imatinib mesylate caused a dose-dependent inhibition of TA (up to 90% at a concentration of 15 *μ*M IM) in c-kit-expressing SK-N-MC (Ewing sarcoma), SK-MEL-28 (melanoma), RPMI 8226 (myeloma), MCF-7 (breast cancer) and HSC 536/N (Fanconi anaemia) cells as well as in ba/F3 (murine pro-B cells), which do not express c-kit, BCR-ABL or PDGF-R. Imatinib mesylate did not affect the activity of other DNA polymerases. Inhibition of TA was associated with 50% inhibition of proliferation. The inhibition of proliferation was associated with a decrease in the S-phase of the cell cycle and an accumulation of cells in the G2/M phase. No apoptosis was observed. Inhibition of TA was caused mainly by post-translational modifications: dephosphorylation of AKT and, to a smaller extent, by early downregulation of hTERT (the catalytic subunit of the enzyme) transcription. Other steps of telomerase regulation were not affected by IM. This study demonstrates an additional cellular target of IM, not necessarily mediated via known tyrosine kinases, that causes inhibition of TA and cell proliferation.

Imatinib mesylate (IM, Gleevec, STI571) belongs to the new generation of rationalised-developed drugs, aimed against specific targets in malignant cells. Its original target was the chimeric protein, BCR-ABL, formed as a result of the molecular juxtaposition of two genes, *BCR* and *ABL*, on a newly created Philadelphia chromosome in chronic myelogenous leukaemia (CML) patients. BCR-ABL is a tyrosine kinase, involved in intracellular signalling pathways leading to proliferation (Capdeville *et al*, 2002).

However, BCR-ABL is not the only protein kinase that is affected by the drug. The kinase activity of other receptors – platelet-derived growth factor receptor (PDGF-R) and the stem-cell factor receptors (c-kit) Abl and Arg – has been shown to be blocked by Gleevec as well. The drug has little effect on other kinases (Kurzrock *et al*, 2003). However, inhibition of other protein kinase receptor autophosphorylation was demonstrated in FLT3, CFMC, c-FMC, v-fms and v-SRC, although at a much higher dosage (IC_50_>10 *μ*M) (Capdeville *et al*, 2002; Kurzrock *et al*, 2003).

Autophosphorylation of these protein kinases initiates signal transduction pathways that control crucial cell functions in tumorigenesis, including proliferation, adhesion, apoptosis and differentiation (Heinrich *et al*, 2002).

c-kit kinase activity is essential for normal haematopoiesis, melanogenesis, gametogenesis, and growth and differentiation of mast cells and interstitial cells of Cajal (ICC) (Heinrich *et al*, 2000). Active c-kit is present in a variety of malignancies such as germ line, mast cells, gastrointestinal stromal (GIST) tumours, small cell lung carcinoma, melanoma, breast cancers, acute myelogenous leukaemia (AML) and neuroblastoma (Heinrich *et al*, 2002). Preclinical studies have shown that c-kit-dependent malignancies such as GIST demonstrate a reduced proliferation rate after IM treatment (Capdeville *et al*, 2002). These findings were corroborated by reports of clinical response to IM treatment.

Until recently, the clinical activity of IM was attributed solely or mainly to its tyrosine kinase inhibitory effect. Two lines of evidence suggest that IM may target other cellular, yet unidentified components: clinical observations show that patients suffering from CML respond to the drug even in the blastic crisis stage of the disease, which is probably caused by other molecular changes (Goldman, 2002). In addition, a recent study describes the inhibitory effect of IM on various multiple myeloma (MM) cell lines. The drug is effective in cell lines expressing c-kit, as well as in those in which the receptor is not expressed (Pandiella *et al*, 2003).

In the light of the important role of telomerase in malignant transformation, we surmised that IM's antineoplastic activity might be associated with telomerase regulation.

Telomeres are specialised nucleoprotein complexes that protect against fusion and degradation of linear chromosomes (Blackburn, 2001). A critical shortening of telomeres, associated with each DNA replication in most normal somatic cells, leads to the cessation of cell division, a metabolic state called senescence (Kim *et al*, 2002). Telomeric dysfunction is associated with very short telomeres, induces cell ‘crisis’ with chromosomal instability, end-to-end chromosome fusions, activation of DNA checkpoint responses, apoptosis and cell death (Blackburn, 2001; Kim *et al*, 2002). Telomerase is a ribonucleoprotein DNA polymerase composed of an RNA template (hTR) and a catalytic protein subunit (hTERT), which is responsible for synthesising the telomeric repeats (TTAGGG)_*n*_ at chromosomal ends to compensate for telomere loss (Kim *et al*, 2002). The proper functioning of telomerase requires several other associated proteins (Aisner *et al*, 2002). The regulation of telomerase is multifactorial, involving transcriptional and post-translational steps including complicated gene promoter consisting of binding sites for a variety of transcription factors, alternative splicing pathways, protein phosphorylation and dephosphorylation processes. Examination of the correlation between telomerase activity (TA) and the expression of hTERT gene or hTR revealed that usually the amount of hTERT transcript is the limiting factor of its activity (Colgin *et al*, 2000). Telomerase post-translational modifications switch the enzyme from an active form (phosphorylated) to an inactive protein (dephosphorylated). Phosphorylation of its tyrosine residues is performed by both PKC*α* and *ς* or by AKT (protein kinase B).

Dephosphorylation is performed by protein phosphatase 2A (PP2A). It has been postulated that phosphorylation and dephosphorylation of telomerase is associated with its translocation from the cytoplasm into the nucleus prior to binding to its telomeric substrate (Aisner *et al*, 2002).

It is well established that telomerase upregulation or activation of alternative mechanisms of telomere maintenance (ALT – alternative lengthening of telomeres) has a major role in malignant transformation. If cells are to survive and proliferate indefinitely, telomere preservation is essential for the immortalisation process (Collins and Mitchel, 2002). Telomerase activity has been demonstrated in high levels in over 85% of all malignancies (Shay *et al*, 2001). Although telomerase is not an oncogene (Harley, 2002), transfection of hTERT into normal epithelial or endothelial cells transformed with SV40 large T antigen and N-ras oncogene allows cells to bypass crisis and ultimately achieve malignancy, confirming the role of telomerase in cellular immortalisation and tumorigenesis (Shay *et al*, 2001).

Therefore, telomerase is considered to be an attractive target in anticancer therapy. Telomerase inhibition by numerous strategies, including dominant-negative telomerase mutants (Hahn *et al*, 1999; Boklan *et al*, 2002), small molecular weight compounds (Seimiya *et al*, 2002), reverse transcriptase inhibitors (Damm *et al*, 2001), G quadruplex interactive agents (Izbicka *et al*, 1999; Grand *et al*, 2002) and antisense oligonucleotides complementary for hTR (Herbert *et al*, 1999, 2002; Akiyama *et al*, 2003; Pang *et al*, 2003), has been shown to limit the growth of immortalised tumour cell lines by inducing progressive telomere shortening and cell death. Moreover, a recent report from Moore's laboratory (Wang *et al*, 2004) describes the inhibition of MM and non-Hodgkin's lymphoma xenografts established from cell lines with short telomeres, which is associated with increased tumour apoptosis.

In parallel, it is possible that some cytotoxic drugs act via the inhibition of telomerase, leading to apoptosis. For example, we have shown that thalidomide downregulates the expression of hTERT gene in MM cells, via intercalation to its promoter region, in GC-rich domains that serve as binding sites for SP1 transcription factor (Drucker *et al*, 2003).

Recently, it has been shown that telomerase inhibition achieved by dominant-negative allele of telomerase (DN-hTERT) sensitises BCR-ABL-positive leukaemia cells to IM (Tauchi *et al*, 2002). However, the effect of IM on TA in c-kit-expressing cell lines has never been explored. Therefore, we examined the response of TA and its regulation with reference to IM application in these settings. Our experimental system includes the SK-N-MC cell line, an Askin's tumour cell line, related to Ewing sarcoma lines as well as several other c-kit-expressing and nonexpressing malignant cell lines.

## MATERIALS AND METHODS

### Cell lines

SK-N-MC (Ewing sarcoma) cell line was kindly provided by Dr Gad Lavie (Sheba Medical Center, Ramat-Gan, Israel). RPMI 8226 (MM cell line) and MCF-7 (breast cancer cell line) were purchased from the ATCC, USA. HSC 536/N (pro-B-lymphocytes Fanconi C cells) was donated by Prof. Ina Fabian (Tel Aviv University, Tel Aviv, Israel). SK-MEL-28, melanoma cells, was given by Dr Britta Hardy (Felsenstein Medical Research Center, Rabin Medical Center, Petah-Tikva, Israel). All cell lines were maintained in RPMI 1640 supplemented with 10–15% heat-inactivated fetal calf serum (FCS), glutamine (2 mM), penicillin and streptomycin (Beit Haemek, Israel). Ba/F3 and WEHI cells were received from Dr Drorit Neuman (Tel Aviv University, Tel Aviv, Israel). Ba/F3 cells were maintained as previously described (Drummond-Barbosa *et al*, 1995) in RPMI 1640 supplemented with 10% heat-inactivated FCS and 10% WEHI conditioned medium as a source of IL-3 and antibiotics.

All cell lines were c-kit positive, except for Ba/F3, which is negative for c-kit, BCR-ABL and PDGF-R.

Proliferation assays, apoptosis analyses and TA assays were performed on all cell lines. SK-N-MC line was chosen for detailed analysis of various mechanisms related to telomerase regulation.

### Proliferation assay

Adherent cells (1 × 10^4^ cells ml^−1^ SK-N-MC, 9 × 10^3^ cells ml^−1^ SK-Mel-28) were seeded in quadruplicate in 24-well plates. Imatinib mesylate (kindly provided by Novartis, NJ, USA) was added at concentrations ranging from 0 to 20 *μ*M. After 3–5 days, cytotoxicity was determined with the sulphorohodamine B assay (Skehan *et al*, 1990). Briefly, cultures were fixed with 10% trichloroacetic acid (Sigma, Israel) and stained with 0.4 (w v^−1^) sulphorhodamine B (Sigma, Israel) dissolved in 1% acetic acid. Unbound dye was removed by four washes with acetic acid (1%), and the dye-stained protein was extracted with unbuffered Tris base (10 mM). The absorbance of stained protein samples was determined in a 96-well microtitre ELISA plate reader (550 nm). The results obtained with the assay were confirmed by counting cells in a haemocytometer. The cytotoxicity of all nonadherent cells (RPMI 8226, HSC 536/N and Ba/F3) was determined using Trypan blue exclusion assay.

### Apoptosis assay

Apoptosis was assessed by detecting DNA fragmentation using the cell death ELISA kit (Roche, Mannheim, Germany). SK-N-MC cells (1 × 10^4^) were plated in each well of 24-well plates. Cells were incubated in the presence of IM (0–15 *μ*M). Each treatment was performed in triplicate. After 5 days, cells were washed once with phosphate-buffered saline, and 0.5 ml lysis buffer was added. After 30 min incubation, the supernatant was removed and assayed for DNA fragments, according to the manufacturer's instructions. The absorbance at 405 and 492 nm was determined in a computer-interfaced 96-well microtitre plate reader. Additional plates, treated as above, were analysed for cell number using the sulphorhodamine B method. The results obtained from the DNA fragmentation assay were then normalised for cell numbers. Results were expressed as relative apoptosis to untreated controls (enrichment factor).

Alternatively, cells defined by FACS analysis in the pre-G1 stage of the cell cycle were considered apoptotic.

### Cell cycle analysis

Cells (0.7–1 × 10^4^ ml^−1^) were cultured for 1–5 days. Floating and adherent cells were combined, washed with PBS and nuclei prepared from 5 × 10^5^ to 1 × 10^6^ cells for flow cytometric analysis using a detergent-trypsin method followed by staining with propidium iodide (Vindelov *et al*, 1983). DNA content was analysed by FACSCALIBUR (Becton Dickinson, San Jose, CA, USA), using ModFitLT cell cycle analysis software (Verity Software House Inc., Topsham, ME, USA).

### Telomeric repeat amplification protocol assay

Measurement of TA was performed by the PCR-based TRAP assay, using the TRAP_EZE_ telomerase detection kit (Intergene, NY, USA), according to the manufacturer's instructions and as described previously (Kim *et al*, 1994). Briefly, isolated cells were incubated with ice-cold CHAPS (3-[(3-cholamidopropyl) dimethylammonio]-1-propane sulphonate) lysis buffer for 30 min at 4°C (1 × 10^6^ cells 100 *μ*l^−1^) and were subsequently centrifuged at 13 000 r.p.m. for 30 min at 4°C. The supernatant was then collected and the protein concentration was determined by the Bradford assay (Bio-Rad Laboratories, CA, USA). Protein extract (0.2 *μ*g) was assayed for TRAP analysis. Each reaction was performed in 50 *μ*l reaction mixture containing 10 × TRAP buffer, dNTP mix, TS primer, TRAP primer mix and *Taq* polymerase. Reactions were performed at 30°C for 30 min and were then subjected to PCR amplification for 30 cycles of 94°C, 59 and 72°C for 30 s each, and were separated by electrophoresis on 12.5% polyacrylamide gels, in a Mighty Small II gel apparatus (Hoffer Scientific Instruments). Gels were stained with SYBER^R^ Green nucleic acid gel stain (Amresco, Ohio, USA). Quantifications were performed using the Quantity-one software for Bio-Rad's Image analysis systems (Bio-Rad Laboratories). Telomerase activity was calculated according to the following formula: TPG (U)=(*X*–*X*_0_)/*C*:(*r*–*r*_0_)/Cr × 100, where TPG is the total product generated, *X* signifies non-heat-treated samples, *X*_0_ signifies heat-treated samples, *C* represents the 36 bp internal PCR control, *r* is the TSR8 quantification control and *r*_0_ is 1 × CHAPS lysis buffer control. All results were determined from at least four to six independent TRAP assays and average activity was calculated.

### RNA purification and RT–PCR analysis for hTERT, PKC*α*, AKT 1–3 and PP2A

Expression of the relevant genes was performed by a semiquantitative multiplex RT–PCR technique. Total RNA was extracted from cells using the Purescript RNA isolation kit (Gentra Systems, MM, USA) according to the manufacturer's instructions. RNA (1 *μ*g) was then reverse transcribed into single-stranded DNA with Superscript™II RNase Reverse Transcriptase (Gibco BRL, England, UK). Each RT–PCR reaction was performed with the genes’ specific primers as well as *β*-actin primers as an internal control. RT–PCR products were separated on 2% agarose gels and the relative intensity of the amplified products was calculated compared to the housekeeping gene, *β*-actin, using the Quantity-one software for Bio-Rad Image analysis systems (Bio-Rad Laboratories).

hTERT mRNA was amplified by PCR using the following primers: forward primer 5′-CGGAAGAGTGTCTGGAGCAA-3′(corresponding to GenBank position 1785–1804, accession number AFO 18167; Poremba *et al*, 2000) and reverse primer 5′-CTCCCACGACGTAGTCCATG-3′(GenBank position 1961–80). Amplification was performed with 28 cycles of 94°C for 30 s, 59°C for 30 s and 72°C for 30 s.

Primers for hTERT alternative splicing were: forward primer 5′-GCCTGAGCTGTACTTTGTCAA-3′ (corresponding to position 2109–2130, GenBank accession number AF128893/4) and reverse primer 5′-AGGCTGCAGAGCAGCGTGGAGAGG-3′ (corresponding to position 2531–2507) (Yi *et al*, 2001). Amplification was performed with 35 cycles of 94°C for 20 s, 60°C for 20 s and 72°C for 40 s.

The *β*-actin primer sequences were: forward primer 5′-GACCACACCTTCTACAATGAG-3′ and reverse primer 5′-GCATACCCGTCGTAGATGGGG-3′.

The PKC*α* primers sequences were: forward primer 5′-CGAGGAAGGAAACATGGAACTCAG-3′ (corresponding to position 908–926, GenBank accession number X53479) and reverse primer 5′-CCTGTCGGCAAGCATCACCTTT-3′ (position 1101–1079) (Oshevski *et al*, 1999). PCR program for PKC*α* was 94°C for 30 s, 57°C for 30 s and 72°C for 30 s.

The AKT 1 primers were: forward primer 5′-ATGAGCGACGTGGCTATTGTGAAG-3′ (corresponding to position 243–267, GenBank accession number AF283818) and reverse primer 5′-GAGGCCGTCAGCCACAGTCTGGATG-3′ (corresponding to positions 116–91).

The AKT 2 primers were: forward primer 5′-ATGAATGAGGTGTCTGTCATCAAAGAAGGC-3′ (corresponding to position 88–117, GenBank accession number M95936) and reverse primer 5′-TGCCTTGAGGCTGTTGGCGACC-3′(corresponding to positions 422–402).

The AKT 3 primers were: forward primer 5′-ATGAGCGATGTTACCATTGT-3′ (corresponding to position 1–20, GenBank accession number NM_005465) and reverse primer 5′-CAGTCTGTCTGCTACAGCCTGGATA-3′ (corresponding to positions 327–303) (Nakatani *et al*, 1999). PCR program for all AKT isoforms was 30 cycles of 94°C for 45 s, 55°C for 45 s and 72°C for 45 s.

The PP2A primers were: forward primer 5′-CCTCTTGTCATCAACAGCCGTG-3′ (corresponding to position 1296–1317, GenBank accession number ak097599) reverse primer 5′-GCAGGAAGAACCCACAAAGTG-3′ (corresponding to position 2056–2037) (Luss H *et al*, 2000). Amplification was carried out with 30 cycles of 94°C for 45 s, 60°C for 45 s and 72°C for 45 s.

All experiments were duplicated and repeated at least three times.

### Western blotting

Phosphorylated AKT was detected in the cells after induction with FCS as follows: cells were grown in RPMI 1640 medium deprived of serum for 24 h. Imatinib mesylate was then added to the culture for 90 min. To induce phosphorylated AKT, FCS was added to the cell cultures for 1 h. Cells were then harvested, washed by PBS and lysed using the TRAP kit CHAPS lysis buffer. Protein concentration was determined using the Bradford assay (Bio-Rad Lab., Hercules, CA, USA). Identical protein amounts of all samples were subjected to PAGE. The AKT protein or the phosphorylated form of AKT protein was detected by specific monoclonal antibody (Cell Signaling, SC, USA) in 1 : 1000 dilution, followed by HRP-conjugated goat anti-rabbit antibody (Jackson Lab., West Grove, PA, USA). Visualisation of both protein expressions was performed by the SuperSignal® West Pico Chemiluminescent Substrate kit (Pierce, IL, USA) according to the supplied protocol. Quantification of the signals was performed by using the Quantity-one software for Bio-Rad Image analysis systems (Bio-Rad Laboratories). Phosphorylated AKT expression was calculated relative to the total signal obtained from the AKT protein.

### Nuclear localisation of hTERT

Localisation of telomerase was analysed in two ways: *in situ* TRAP assay and immunofluorescence. *In situ* TRAP assay was performed as described previously (Ohyashiki *et al*, 1997). Cells were mounted on silane-coated slides and subjected to TA reaction in the presence of 20 mM Tris-HCl (pH 8.3), 1.5 mM MgCl_2_, 63 mM KCl, 0.05% Tween 20, 1 mM EGTA, 50 *μ*M deoxynucleoside triphosphatase, bovine serum albumin (BSA) (0.1 mg ml^−1^), 1 *μ*g T4 gene protein (Boehringer Mannheim, Germany), 2 U *Taq* polymerase and 10 pmol FITC-labelled TS forward primer (5′-AATCCGTCGAGCAGAGTT-3′; Metabion, Germany). Slides were incubated for 30 min at 22°C in the dark. After TS extension, 25 *μ*M of the same solution was mixed with 10 pmol of FITC-labelled CX reverse primer (5′-CCCTTACCCTTACCCTTACCCTTA-3; Metabion, Germany). Samples were then heated to 90°C for 1.5 min to inactivate the telomerase, and then amplified in PCR thermal cycler (Eppendorf). PCR conditions were 30 cycles of 94°C for 30 s, 50°C for 30 s and 72°C for 90 s. Slides were washed with PBS, sealed with mounting medium containing DAPI (Sigma, Israel) for nucleus counterstain and observed under a fluorescence microscope (Olympus). The presence of FITC dots in the nucleus reflected TA.

For immunofluorescence analyses, cells were grown in Lab-Tek Chamber Slides™ system (Nalge Nunc International Corp., USA), washed and fixed with PFA buffer (4% paraformaldehyde in PBS with 0.5% Triton X-100) for 30 min. Cells were then washed with 0.5% Triton X-100 in PBS and blocked with 4% BSA and 0.5% Triton X-100 in PBS for 30 min. Subsequently, cells were incubated with 1 : 20 antitelomerase antibody (mouse monoclonal antibodies, Novocastra Laboratories, England, UK) in blocking buffer for 2 h.

After washing with PBS, cells were incubated in blocking buffer containing FITC (goat anti-mouse fluorescein conjugated, Chemicon International, CA, USA) as secondary antibody (1 : 20) and anti-nucleolin antibody (c23, Santa Cruz Biotechnology, CA, USA) conjugated to TRITC (1 : 10) for 30 min. Cells were stained with DAPI in a mounting medium (Vector Laboratories, England, UK) to counterstain the nucleus. An Olympus microscope with appropriate filters was used. Images were collected and processed using Olympus DP-software.

### DNA polymerisation reaction

The nuclear fractions were tested for DNA synthesis with defined DNA–DNA template primers with the following sequence:

5′-ATTTCACATCTGACTT-3′

3′-CCTAAAGTGTAGACTGAATTGTTTGGCGGA-5′

The primer was end-labelled at the 5′ end with T4 polynucleotide kinase (purchased from MBI, Fermentas, Germany) and [*γ*-^32^P]ATP. The end-labelled primer was annealed to the template DNA as described (Bakhanashvili and Hizi, 1992).

The DNA polymerisation reactions contained 10 mM Tris-HCl (pH 7.5), 2 mM DTT, 10 mM MgCl_2_, 100 *μ*M dNTP (Pharmacia Biotech, Uppsala, Sweden), 0.1 mg ml^−1^ BSA and 5′-end-labelled DNA/DNA template-primer. The reaction was started by the addition of 10 *μ*g nuclear extract. Assays were carried out at 37°C for 10 min. Aliquots (5 *μ*l) were removed into 5 *μ*l of formamide dye mix, denatured at 100°C for 5 min and cooled on ice. Electrophoretic analyses were performed in 16% polyacrylamide sequencing gels followed by autoradiography. The results were reproduced three times using separate preparations of nuclear extracts.

## RESULTS

### Effect of IM on the proliferation of SK-N-MC cells

**Figure 1:**
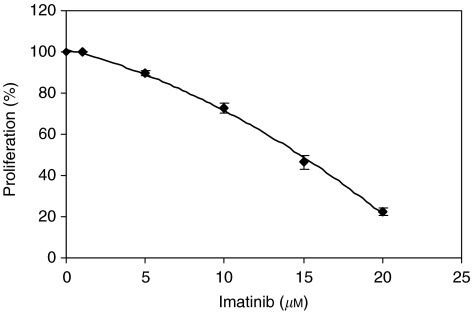
Growth inhibition of SK-N-MC cells by imatinib after 5 days. Cells were grown in the presence of imatinib at 0–20 *μ*M for 5 days and their proliferation was calculated by the SRB method. The graph represents five independent experiments conducted in duplicate.

The effect of IM on cell growth was determined using the SRB assay. Figure 1 describes a dose-dependent growth inhibitory effect. A 50% reduction in cell numbers was achieved by 15 *μ*M IM administration. Therefore, concentration of 15 *μ*M was chosen for further experiments.

### Apoptotic effect of IM on SK-N-MC cells

In order to determine whether imatinib has an apoptotic effect on SK-N-MC cells, we analysed them for DNA fragmentation using the cell death detection ELISA method. Cells exposed to IM at 10 and 15 *μ*M demonstrated enrichment factor values of 119 and 128.5, respectively, compared to 100 as controls, which correspond to less than 10% apoptosis. These values suggest that IM treatment had no significant apoptotic effect on SK-N-MC cells. Similar results were obtained when pre-G1 phase of the cell cycle was analysed by FACS (not shown).

### Cell cycle analysis

To explore whether IM exerts its antiproliferative effect via cell cycle arrest, we determined the cell cycle status of the treated cells, compared to the controls. Imatinib mesylate caused changes in cell cycle status, which were most prominent in days 4 and 5. As shown in Table 1, IM treatment resulted in the arrest of cells in G2/M phase of the cell cycle. This arrest was accompanied by a decrease in the accumulation of cells in the S phase.

### Telomerase activity levels after IM treatment

**Figure 2:**
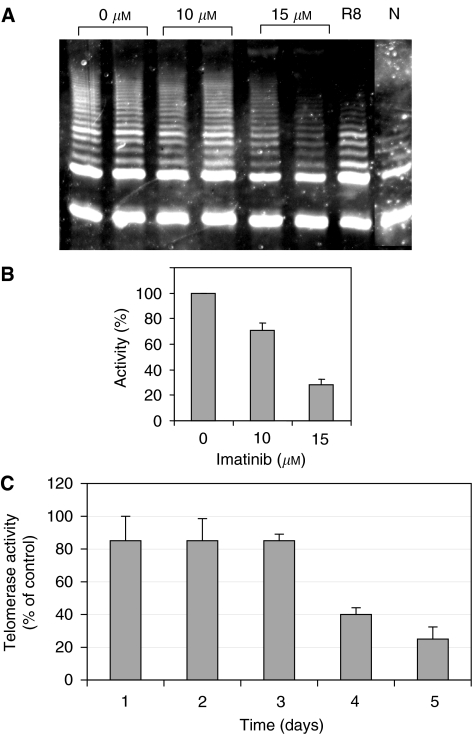
Telomerase activity in SK-N-MC cells treated with imatinib. (**A**) Telomeric repeat amplification protocol assay describing TA (one representative experiment). Imatinib concentrations are indicated above. R8: internal PCR control; N: negative control. (**B**) Quantification of TA affected by 15 *μ*M imatinib (average of four independent assays). (**C**) Kinetics of TA affected by imatinib.

SK-N-MC Ewing sarcoma cells were exposed for 5 days to 10–15 *μ*M IM, the concentration shown to cause 35–65% growth inhibition, respectively. Telomerase activity was assessed using the TRAP assay, as described in Materials and Methods. The activity of telomerase was reduced in a dose-dependent manner and compared to its activity in the untreated control cells (Figure 2A and B). Imatinib mesylate at 10 *μ*M led to 35% TA inhibition, whereas 15 *μ*M caused about 75% inhibition. To examine whether the downregulation of TA was a nonspecific result of reduced proliferation, highly enriched nuclear fractions of treated and nontreated cells were analysed for DNA polymerisation activity of DNA polymerase *α,* a major nuclear enzyme. The results obtained revealed that there was no decrease in DNA polymerase capacity of the nuclear enzyme (not shown), thus indicating a specific effect of the drug on TA. Figure 2C shows the gradual effect of IM on TA over time. In the first 3 days after the addition of the drug, the activity of the enzyme was reduced to about 85% compared to controls. After 4 days, only 50% of the initial TA was detected. The inhibition reached its maximal level after 5 days, when approximately 80% of TA was abolished.

To clarify whether telomerase inhibition is mediated by direct interaction of the drug with the enzyme, we added IM at 15 *μ*M to SK-N-MC cell lysates 30 min prior to TRAP assay. Imatinib mesylate had no effect on TA in these settings (not shown).

### Regulation of hTERT

Telomerase is known to be regulated both transcriptionally and post-translationally (Aisner *et al*, 2002).

#### Transcriptional regulation of hTERT

hTERT expression is regulated by the activity of its promoter, modulated by numerous transcription factors and by alternative splicing. We analysed telomerase expression at both levels.

Expression of hTERT total mRNA: We determined the expression of hTERT 24, 48, 72 and 96 h following cellular exposure to IM. The expression of the gene was evaluated relative to the expression of the *β*-actin, a housekeeping gene.

**Figure 3:**
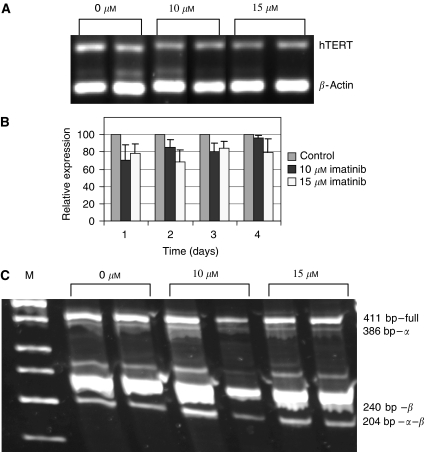
Transcription regulation of hTERT in SK-N-MC cells exposed to imatinib. hTERT expression was evaluated by RT–PCR, using primers homologous to the total gene transcript. The alternative spliced variants were amplified using primers homologous to the two splice sites of the gene mRNA molecule, namely *α* and *β*. (**A**) An example of hTERT total transcript expression (after 72 h of imatinib treatment). (**B**) Quantification of the hTERT expression (an average of four experiments). (**C**) Alternative splicing of hTERT (after 72 h of imatinib treatment). Sizes of the various splice forms are indicated on the right. M: molecular size marker.

Analyses of hTERT mRNA levels in treated cells compared to untreated cells by RT–PCR revealed a consistent decrease in its expression. The decrease ranged between 15 and 30% and was similar at 10 and 15 *μ*M concentrations, as well as at all time points (Figure 3A and B).

Alternative splicing of hTERT: hTERT gene possesses six different alternative splicing forms, in which four contain deletions and two contain insertions of the gene. Only the full intact transcript can be translated into a properly active enzyme. Examination of hTERT alternative splicing forms showed that there was no difference between the various transcripts in the treated compared to the nontreated cells (Figure 3C). The analysis was carried out using primers that are complementary to the two regions of deletions (named *α* and *β*), to amplify four expected alternative forms of hTERT transcript.

#### Post-translational modification of telomerase

The other level of telomerase regulation is post-translational. The enzyme becomes active upon phosphorylation, but inactive following dephosphorylation. We analysed the expression of the enzymes known to be involved in these modifications.

**Figure 4:**
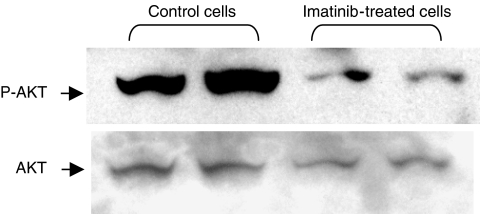
Phosphorylated AKT expression in response to imatinib treatment. Phosphorylated AKT and total AKT protein levels after SK-N-MC exposure to imatinib were analysed by Western blotting, as described in Materials and Methods.

Phosphorylation by PKC*α* and AKT: Telomerase is phosphorylated by the two kinases AKT and PKC*α*. To determine whether its enzymatic activity was inhibited through modulation of its phosphorylation status, the possible direct effect of IM treatment on the phosphorylated form of AKT was monitored by Western blot analysis on SK-N-MC-treated cells using specific AKT or phospho-AKT antibodies. As can be seen in Figure 4, the amount of phosphorylated AKT decreased dramatically after exposing the cells to 15 *μ*M IM for 90 min. Imatinib mesylate caused 72% reduction in the phosphorylated form of AKT.

**Figure 5:**
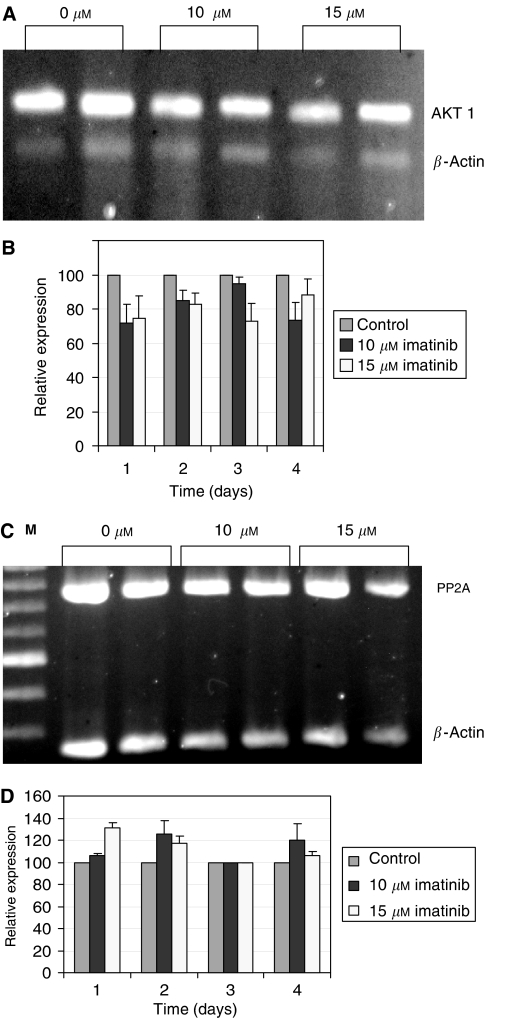
hTERT post-translational modifications: expression of hTERT kinase, AKT 1, and hTERT phosphatase, PP2A, in response to imatinib treatment at various concentrations. The expression of both genes was monitored by RT–PCR. (**A**) Levels of AKT 1 mRNA in response to various concentrations of imatinib. (**B**) Quantification of AKT 1 mRNA. (**C**) Expression of PP2A analysed by RT–PCR (an example of expression after 3 days is shown). M: molecular weight marker. (**D**) Quantification of PP2A expression (an average of four independent experiments is shown).

In addition, the expression of the genes encoded for AKT and PKC*α* was monitored. The AKT kinases appear in three forms: AKT 1, 2 and 3. RT–PCR revealed no changes in the expression of AKT 2 or AKT 3 (not shown). However, there was a tendency for expression of AKT 1, the third AKT form, to be inhibited (Figure 5A and B).

The average decrease in AKT 1 expression was about 25%.

RT–PCR analyses of PKC*α* expression showed no change in its expression in response to IM treatment (not shown).

Dephosphorylation by PP2A: As TA is dependent on its phosphorylation status, dephosphorylation abolishes its activity. The expression of PP2A in SK-N-MC cells exposed to IM was followed by RT–PCR. Increase in its expression was seen mainly 24 and 48 h after IM treatment. However, this increase was reduced after 72 and 96 h of IM exposure, compared to the expression of the control housekeeping gene in the nontreated cells (Figure 5C and D).

### Subnuclear localisation of telomerase

**Figure 6:**
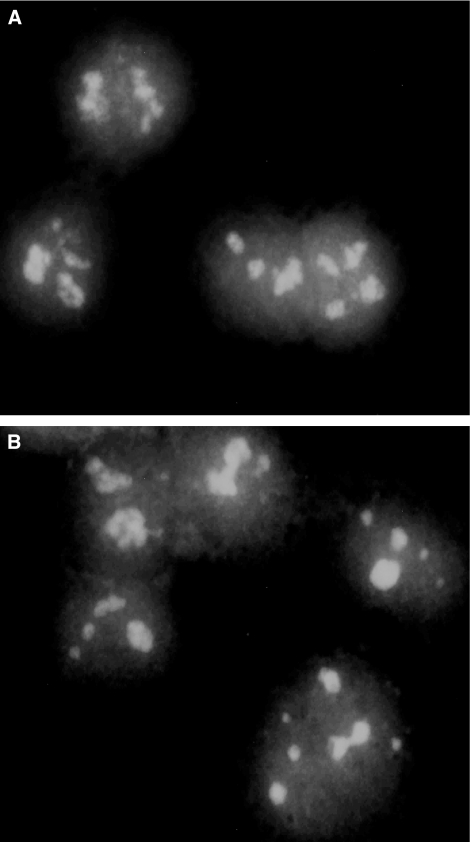
Subnuclear localisation of hTERT in response to imatinib. SK-N-MC cells were exposed to 15 *μ*M imatinib for 5 days, and stained with FITC-conjugated anti-telomerase antibodies as described in Materials and Methods. (**A**) Control cells. (**B**) Cells treated with imatinib.

Recently, Collins *et al* reported another important aspect of telomerase regulation (Wong *et al*, 2002). In their study, they showed the significance of the telomerase subnuclear localisation, which affects its activity. Localisation of the enzyme in the nucleolus, away from its natural telomeric substrate, results in a nonactive telomerase. Its activity is restored when it is in the nucleoplasm, close to the telomeres. Therefore, we determined its localisation after IM treatment. Localisation of telomerase was examined in two different ways: firstly, by *in situ* TRAP assay, using fluorescent primer as its substrate and, secondly, by double staining of telomerase and nucleolin, a protein that is typical of nucleoli. There was no difference in the localisation of telomerase between treated and nontreated cells, as its presence was detected in the nucleoli as well as in the whole nucleoplasm (Figure 6).

### Effect of IM on growth and TA in other cell lines

**Figure 7:**
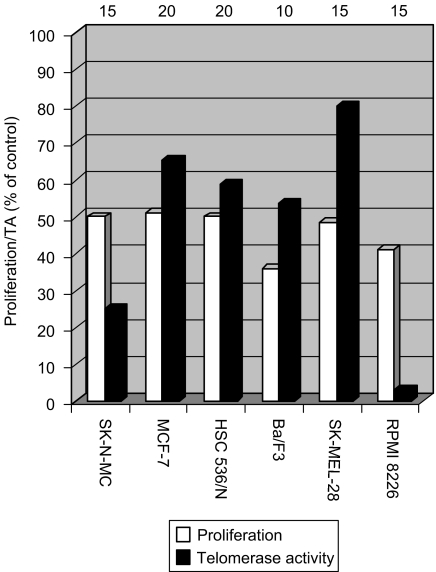
Imatinib inhibits proliferation and TA in various cell lines. Different cell lines were exposed to imatinib to achieve approximately 50% growth inhibition. Proliferation and TA were evaluated as described in Materials and Methods. For each cell line, the first bar represents the proliferation, and the second bar describes TA. Both values are expressed as percentages of values in control cells. Imatinib concentrations used are shown above each pair of bars in *μ*M.

The following cell lines expressing c-kit were tested for their growth response to the drug: HSC 536/N, pro-B-lymphocytes Fanconi C cells; RPMI 8226, MM cells; SK-MEL-28, melanoma cells; MCF-7, breast cancer cell line. All the cells expressing c-kit responded to IM at the same range of dosage, 10–20 *μ*M. The drug caused about 50% decrease in proliferation, and a differential inhibition of TA (Figure 7). In one cell line, SK-MEL-28, the native TA in control cells was low compared to the other cell lines, and the effect of the drug on its activity was much less pronounced (inhibition of about 20%). The latter's effect may be correlated with the low level of TA in these cells, reflecting a lesser proliferative dependency on TA.

**Figure 8:**
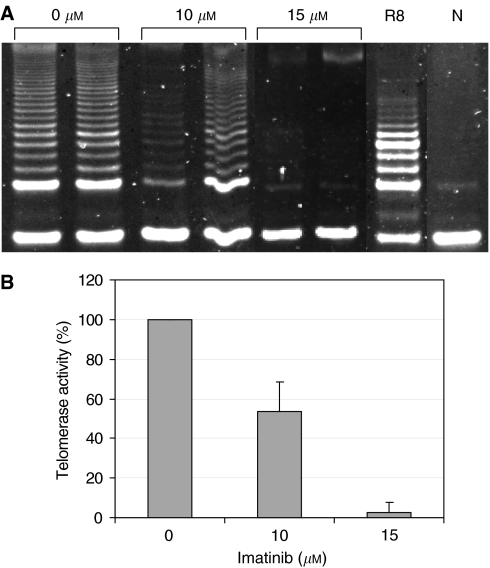
Telomerase activity in cells not expressing c-kit. Ba/F3 cells were exposed to imatinib at the indicated concentrations for 3 days, and TA was analysed by the TRAP assay. (**A**) An example of TRAP assay results. R8: internal standard of the TRAP assay; N: negative control with no cell extract. (**B**) Quantification of telomerase inhibition by imatinib. Telomerase activity in the treated cells is shown as a percentage of control values.

We then explored the effect of IM on pro-B cell line, Ba/F3, which does not express any of the known tyrosine kinase receptors (BCR-ABL, c-kit or PDGFR). Treatment with 10 *μ*M IM for 3 days resulted in the inhibition of proliferation (65%) and TA (50%). Administration of 15 *μ*M IM for 5 days reduced the proliferation and TA by 50% and more than 95%, respectively (Figure 8). These data suggest that the downregulation of TA caused by IM is not necessarily c-kit dependent.

## DISCUSSION

Telomerase is an unusually challenging target for anticancer drug development as it is intrinsic to the proliferation capacity of cancer cells. Over 90% of tumour cells express active telomerase, yet somatic cells rarely possess its activity. Due to its remarkable importance in the biology of malignancy, the effects of various drugs on its activity are of potential importance.

Our results demonstrate the ability of IM to inhibit TA in cell lines independently of c-kit expression. More specifically, a marked decrease of TA was demonstrated in SK-N-MC cells (Ewing sarcoma cells), as well as other cell lines expressing c-kit including HSC 536/N (Fanconi anaemia cells), SK-MEL-28 (melanoma cells), RPMI 8226 (MM cells) and MCF-7 (breast cancer cells). Telomerase activity was also inhibited to the same extent in ba/F3 (murine pro-B cells), in which the c-kit receptor (or BCR-ABL or PDGF-R) is not expressed. This inhibitory effect was specific to telomerase, as the drug did not affect a different DNA polymerase, DNA polymerase *α*.

The effect of IM on cell proliferation was quite similar in cells expressing or nonexpressing c-kit. The drug inhibited 50% of cellular proliferation at a concentration of about 15 *μ*M. This is in agreement with a previous study, which demonstrates the same dosage response of 10–15 *μ*M for other Ewing sarcoma cell lines (Merchant *et al*, 2002). Notably, MM cells responded to the drug at similar concentrations (Pandiella *et al*, 2003). BCR-ABL-positive human leukaemia cell lines such as K562, KU812, MC-3, NMBA-1, KBM-5, Z-33, Z-119, Z-181, human glioma cells (U-87, U-343) and human GIST (GIST882) are more sensitive to IM (Capdeville *et al*, 2002). It seems likely therefore that the antiproliferative effect of IM is tumour specific.

Moderate changes in the cell cycle were detected: a decrease of S phase of the cell cycle accompanied by a partial cellular accumulation in G2/M phase. Interestingly, similar effects on the cell cycle were also reported by others, differing according to the receptor involved in the drug response. A decrease in the proliferation of breast cancer cell lines was associated with a growth arrest at G2/M phase of the cell cycle, a signal that was probably mediated by c-kit (Roussidis *et al*, 2004). Two other reports described a different effect of IM on cell cycle – accumulation of cells mainly in G0/G1 phase – in haematological cell lines expressing BCR-ABL (Nishimura *et al*, 2003; Yin *et al*, 2004).

Recent studies have indicated that telomerase plays a key role in cellular resistance to apoptosis (Fu *et al*, 1999). Interestingly, IM treatment did not induce apoptosis in all cell lines tested in our study. This finding is in accordance with a recent study conducted on K-562, a BCR-ABL-positive cell line, exposed to IM. The authors demonstrated that IM induced a caspase-independent, necrosis-like cell death mediated by the serine protease activity of Omi/HtrA2 (Okada *et al*, 2004). Other studies demonstrated an inhibitory effect of IM on PDGF-R-expressing cell lines, suggesting that this effect was mediated mainly through promoting growth arrest rather than apoptosis (Kilic *et al*, 2000). Although most of telomerase inhibition strategies resulted in decreased proliferation and apoptosis or cell cycle arrest (Hahn *et al*, 1999; Herbert *et al*, 1999; Heinrich *et al*, 2000; Kilic *et al*, 2000; Boklan *et al*, 2002; Seimiya *et al*, 2002; Zaffaroni *et al*, 2002), other studies showed that inhibition of telomerase was accompanied mainly by the inhibition of cell proliferation rather than cell death. For example, hTR antisense inhibited TA in human pancreatic carcinoma cell line, concomitantly with a significant decrease in proliferation, without a crisis or senescence phenomenon (Teng *et al*, 2002; Teng and Fahey, 2002). In another study, histone deacetylase inhibitors, which suppress hTERT expression in prostate cancer cells, also inhibited cell proliferation inhibition with no cell cycle arrest, apoptosis or cell differentiation (Suenaga *et al*, 2002). The results of our study show that IM inhibits proliferation by cell cycle arrest and not by apoptosis.

Telomerase activity is regulated on multiple levels (Cong *et al*, 2002). Its regulation includes transcription, mRNA splicing, maturation and modifications of hTR and hTERT, transport and subcellular localisation of each component, assembly of the holoenzyme to an active ribonucleoprotein, accessibility and proper function on its telomeric substrates (Cong *et al*, 2002). Our results show that IM downregulates TA by two separate mechanisms, operating at different time points. The most prominent effect was a marked dephosphorylation of the AKT protein, which is one of the major kinases that phosphorylate telomerase in the cell. The other kinase that may phosphorylate telomerase is PKC*α* or PKC*ξ*. Although we did not test the levels of phosphorylated PKC in our study, it is reasonable to suppose that the drug does not affect PKC phosphorylation. Initially, IM was selected as a PKC inhibitor. However, modifications on the original compound abolished its inhibitory activity against PKC completely (Bakalova *et al*, 2003). Imatinib mesylate cellular effects are mediated specifically via downregulation of phosphorylation forms of AKT (Heinrich *et al*, 2000) as well. Our results are in keeping with the known mechanism of effects of IM on intracellular signal transudation pathways. hTERT expression was reduced up to 30% after IM treatment, which is considered to be moderate. There was no change in the alternative splicing patterns of hTERT expression as a result of IM treatment. Post-translational changes of telomerase were also monitored indirectly, by evaluating the expression of the enzymes involved in telomerase phosphorylation and dephosphorylation. RT–PCR analysis revealed a slight decrease in AKT 1, which phosphorylates telomerase, and no changes in the other forms of AKT expression or PKC*α*. The expression of PP2A, which deactivates telomerase by dephosphorylation, was increased by 20–30% in treated cells compared to untreated. Together, these results suggest that IM reduced TA mainly via its post-translational modification – a decrease in the phosphorylation form of the enzyme. Recently, another way of telomerase regulation was demonstrated, concerning its subnuclear localisation (Wong *et al*, 2002). The authors showed that the enzyme activity corresponds to its localisation in the nucleoplasm, probably close to its telomeric substrates. Upon inactivation, it is sequestered into the nucleoli. No difference in telomerase subnuclear localisation between treated and nontreated cells was found in this study.

We detected two phases of TA inhibition. Immediately after the drug addition, there was a moderate decrease (∼15%), which lasted 3 days. At days 4 and 5, the inhibition reached its maximal level, that is, 75–80%. These results attest to multiple levels of telomerase inhibition. Decreased expression of telomerase post-translational modifying enzymes (AKT 1) and the slight increase in telomerase dephosphorylating enzyme, PP2A, should be effective during 24 h after drug addition. However, downregulation of the phosphorylated form of AKT, which will eventually decrease the phosphorylated form of telomerase, should be detected only after 3 days, due to the long half-life of the enzyme. Similarly, inhibition of transcription may also be detected at a similar time point, by the same reason. Therefore, we hypothesise an accumulative effect on telomerase regulation in these settings: mainly post-translational modification and also a minor transcriptional regulation.

Our results support other studies, establishing possible links between TA and c-kit, which is via the ERK signal transduction pathway. Preclinical studies have established that IM blocks c-kit autophosphorylation, as well as SCF-mediated downstream cascades such as activation of ERK1, ERK2 and AKT, all of which belong to the MAP kinase family (Buchdunger *et al*, 2000; Heinrich *et al*, 2000). The effect of IM on non c-kit-expressing cells suggests that telomerase inhibition may be mediated by other mechanisms as well. Support for the notion comes from a recent study describing a panel of pancreatic cell lines, all of which are responsive to IM, in a nondependent c-kit, PDGF-R or BCR-ABL pathways. The authors show that c-kit and PDGF-R do exist in these cells, but the response to IM was mediated by other yet unknown pathways (Li *et al*, 2003). Our future work is directed around this issue, in order to clarify possible mechanism(s) by which IM downregulates TA in cells, lacking the known tyrosine kinase targets of IM.

Telomerase activity in general is associated with cellular proliferation. Inhibition of telomerase may lead to proliferation arrest in two ways: by eroding telomeres, which will eventually become too short to allow proper cellular division, and by a more direct effect on the telomere distal end, in which the enzyme no longer protects, thereby leading to immediate proliferation arrest. Shortening of telomeres as a result of different telomerase inhibitors leading to cellular senescence and apoptosis has been demonstrated in several reports (Hahn *et al*, 1999; Herbert *et al*, 1999, 2002; Izbicka *et al*, 1999; Damm *et al*, 2001; Boklan *et al*, 2002; Grand *et al*, 2002; Seimiya *et al*, 2002; Pang *et al*, 2003). The other protective function of telomerase, proposed by Blackburn and her group, was named ‘telomeres capping’ (Chan and Blackburn, 2002). A concomitant inhibition of telomerase and alteration in the expression of AKT 1 and PP2A were detected 24 h following IM treatment. The proliferation arrest observed in our study might be mediated by disrupting the capping ability of telomerase, thus exposing the telomeres with no protection, leading to proliferation arrest. Alternatively, these two processes, telomerase inhibition and cellular senescence, may act in parallel. The variable telomerase inhibition accompanied by about 50% inhibition of cellular proliferation in the various cell lines may support these dynamics. Accordingly, dissociation of TA and proliferation was demonstrated in human HaCaT skin keratinocytes, in which TGF-*β*1 downregulated c-*myc*, TA and cellular proliferation. Overexpression of hTERT in these settings restored TA but not proliferation (Cerezo *et al*, 2002).

Since TA is typical of over 90% of human tumours, and is absent in most somatic cells, its inhibition represents a novel therapeutic strategy. This inhibitory effect may be enhanced by their combination with other conventional cytotoxic drugs (Wang *et al*, 2004). Two concerns have been mentioned regarding telomerase inhibition. Firstly, it may result in damage to normal stem cells. However, the length of telomeres in these cells compared to malignant cells may protect them from that apparent damage. Secondly, inhibiting TA might result in genomic instability, thereby leading to increased neoplasia (Artandi *et al*, 2000). This idea is based on the increased neoplasia in mTR−/− knockout mice, mostly in P53 mutant cells. However, that risk might not be relevant since mice telomere dynamics is significantly different from that of humans (Wright and Shay, 2000).

In summary, we hereby demonstrate an additional mechanism relating telomerase inhibition and reduced cell proliferation, not necessarily mediated via the known tyrosine kinase targets of IM.

## Figures and Tables

**Table 1:** Cell cycle analysis after treatment with imatinib

**Time (days)**	**Treatment**	**%G_0_/G_1_**	**%S**	**%G_2_/M**
4	Control	60.33±2.3	35.52±4.4	3.26±4.3
	15 *μ*M IM	58.01±2.2	26.88±2.5*	15.1±1.1*
5	Control	60.28±4.9	28.7±3.9	9.5±1.6
	15 *μ*M IM	68.35±3.0*	22.13±3.6*	11.95±1.8*

IM=imatinib mesylate.

* *P*-value <0.05.

## References

[bib1] Aisner DL, Wright WE, Shay JW (2002) Telomerase regulation: not just flipping the switch. Curr Opin Genet Dev 12: 80–85 11790559 10.1016/s0959-437x(01)00268-4

[bib2] Akiyama M, Hideshima T, Shammas MA, Hayashi T, Hamasaki M, Tai YT, Richardson P, Gryaznov S, Munshi NC, Anderson KC (2003) Effects of oligonucleotide N3′ → P5′ thio-phosphoramidate (GRN163) targeting telomerase RNA in human multiple myeloma cells. Cancer Res 63: 6187–6194 14559802

[bib3] Artandi SE, Chang S, Lee SL, Alson S, Gottlieb GJ, Chin L, DePinho RA (2000) Telomere dysfunction promotes non-reciprocal translocations and epithelial cancers in mice. Nature 406: 641–645 10949306 10.1038/35020592

[bib4] Bakalova R, Ohba H, Zhelev Z, Ishikawa M, Shinohara Y, Baba Y (2003) Cross-talk between Bcr-Abl tyrosine kinase, protein kinase C and telomerase – a potential reason for resistance to Glivec in chronic myelogenous leukaemia. Biochem Pharmacol 66: 1879–1884 14599545 10.1016/j.bcp.2003.06.001

[bib5] Bakhanashvili M, Hizi A (1992) Fidelity of the reverse transcriptase of human immunodeficiency virus type 2. FEBS Lett 306: 151–156 1378791 10.1016/0014-5793(92)80988-s

[bib6] Blackburn EH (2001) Switching and signaling at the telomere. Cell 106: 661–673 11572773 10.1016/s0092-8674(01)00492-5

[bib7] Boklan J, Nanjangud G, MacKenzie KL, May C, Sadelain M, Moore MA (2002) Limited proliferation and telomere dysfunction following telomerase inhibition in immortal murine fibroblasts. Cancer Res 62: 2104–2114 11929832

[bib8] Buchdunger E, Cioffi CL, Law N, Stover D, Ohno-Jones S, Druker BJ, Lydon NB (2000) Abl protein-tyrosine kinase inhibitor STI 571 inhibits *in vitro* signal transduction mediated by c-kit and platelet-derived growth factor receptors. J Pathol Exp Ther 295: 139–145 10991971

[bib9] Capdeville R, Buchdunger E, Zimmermann J, Matter A (2002) Glivec (STI 571, Imatinib), a rationally developed, targeted anticancer drug. Nat Rev 1: 493–502 10.1038/nrd839 12120256

[bib10] Cerezo A, Kalthoff H, Schuermann M, Schafer B, Boukamp P (2002) Dual regulation of telomerase activity through c-*myc* dependent inhibition and alternative splicing of hTERT. J Cell Sci 115: 1305–1312 11884529 10.1242/jcs.115.6.1305

[bib11] Chan SW-L, Blackburn EH (2002) New ways not to make ends meet: telomerase, DNA damage proteins and heterochromatin. Oncogene 21: 553–563 11850780 10.1038/sj.onc.1205082

[bib12] Colgin LM, Wilkinson C, Englezou A, Kilian A, Robinson MO, Reddel RR (2000) The hTERT alpha splice variant is a dominant negative inhibitor of telomerase activity. Neoplasia 2: 426–432 11191109 10.1038/sj.neo.7900112 PMC1507985

[bib13] Collins K, Mitchel JR (2002) Telomerase in the human organism. Oncogene 21: 564–579 11850781 10.1038/sj.onc.1205083

[bib14] Cong Y-S, Wright WE, Wright Shay JW (2002) Human telomerase and its regulation. Microbiol Mol Biol Rev 66: 407–425 12208997 10.1128/MMBR.66.3.407-425.2002 PMC120798

[bib15] Damm K, Hemmann U, Garin-Chesa P, Hauel N, Kauffmann I, Priepke H, Niestroj C, Daiber C, Enenkel B, Guilliard B, Lauritsch I, Muller E, Pascolo E, Sauter G, Pantic M, Martens UM, Wenz C, Lingner J, Kraut N, Rettig WJ, Schnapp A (2001) A highly selective telomerase inhibitor limiting human cancer cell proliferation. EMBO J 20: 6958–6968 11742973 10.1093/emboj/20.24.6958 PMC125790

[bib16] Drucker L, Uziel O, Tohami T, Shapiro H, Radnay J, Yarkoni S, Lahav M, Lishner M (2003) Thalidomide down-regulates transcript levels of GC-rich promoter genes in multiple myeloma. Mol Pharmacol 64: 415–420 12869646 10.1124/mol.64.2.415

[bib17] Drummond-Barbosa DA, Vaillancourt R, Kazlauskas A, DiMaio D (1995) Ligand-independent activation of the platelet-derived growth factor beta receptor: requirements for bovine papillomavirus E5-induced mitogenic signaling. Mol Cell Biol 5: 2570–2581 10.1128/mcb.15.5.2570 PMC230487 7739538

[bib18] Fu W, Begley JG, Killen MW, Mattson MP (1999) Anti-apoptotic role of telomerase in pheochromocytoma cells. J Biol Chem 274: 7264–7271 10066788 10.1074/jbc.274.11.7264

[bib19] Goldman J (2002) CML yields a few more clues. Blood 99: 3491–3492

[bib20] Grand CL, Han H, Munoz RM, Weitman S, Von Hoff DD, Hurley LH, Bearss DJ (2002) The cationic porphyrin TMPyP4 down-regulates c-MYC and human telomerase reverse transcriptase expression and inhibits tumor growth *in vivo*. Mol Cancer Ther 1: 565–573 12479216

[bib21] Hahn WC, Stewart SA, Brooks MW, York SG, Eaton E, Kurachi A, Bejjersbergen RL, Knoll JHM, Meyerson M, Weinberg RA (1999) Inhibition of telomerase limits the growth of human cancer cells. Nat Med 5: 1164–1170 10502820 10.1038/13495

[bib22] Harley C (2002) Telomerase is not an oncogene. Oncogene 21: 494–502 11850774 10.1038/sj.onc.1205076

[bib23] Heinrich MC, Rubin BP, Longley BJ, Fletcher JA (2002) Biology and genetic aspects of gastrointestinal stromal tumors: KIT activation and cytogenetic alterations. Hum Pathol 33: 484–495 12094373 10.1053/hupa.2002.124124

[bib24] Heinrich MC, Griffith DJ, Drucker BJ, Wait CL, Ott KA, Zigler AJ (2000) Inhibition of c-kit receptor tyrosine kinase activity by STI 571, a selective tyrosine kinase inhibitor. Blood 98: 925–932 10910906

[bib25] Herbert B, Pitts AE, Baker SI, Hamilton SE, Wright WE, Shay JW, Corey DR (1999) Inhibition of human telomerase in immortal human cells leads to progressive telomere shortening and cell death. Proc Natl Acad Sci USA 96: 14276–14281 10588696 10.1073/pnas.96.25.14276 PMC24427

[bib26] Herbert BS, Pongracz K, Shay JW, Gryaznov SM, Shea-Herbert B (2002) Oligonucleotide N3′ P5′ phosphoramidates as efficient telomerase inhibitors. Oncogene 21: 638–642 11850790 10.1038/sj.onc.1205064

[bib27] Izbicka E, Wheelhouse RT, Raymond E, Davidson KK, Lawrence RA, Sun D, Windle BE, Hurley LH, Von Hoff DD (1999) Effects of cationic porphyrins as G-quadruplex interactive agents in human tumor cells. Cancer Res 59: 639–644 9973212

[bib28] Kilic T, Alberta JA, Zdunek PR, Acar M, Iannarelli P, O’Reilly T, Buchdunger E, Black PM, Stiles CD (2000) Intracranial inhibition of platelet-derived growth factor-mediated glioblastoma cell growth by an orally active kinase inhibitor of the 2-phenylaminopyrimidine class. Cancer Res 60: 5143–5150 11016641

[bib29] Kim NW, Piatyszek MA, Prowse KR, Harley CB, West MD, Ho PL, Coviello GM, Wright WE, Weinrich SL, Shay JW (1994) Specific association of human telomerase activity with immortal cells and cancer. Science 266: 2011–2015 7605428 10.1126/science.7605428

[bib30] Kim S, Kaminker P, Campisi J (2002) Telomeres, aging and cancer: in search of a happy ending. Oncogene 21: 503–511 11850775 10.1038/sj.onc.1205077

[bib31] Kurzrock R, Kantarjian HM, Druker BJ, Talpaz M (2003) Philadelphia chromosome-positive leukemias: from basic mechanisms to molecular therapeutics. Ann Intern Med 138: 819–830 12755554 10.7326/0003-4819-138-10-200305200-00010

[bib32] Li J, Kleeff J, Guo J, Fischer L, Giese N, Buchler MW, Friess H (2003) Effects of STI571 (gleevec) on pancreatic cancer cell growth. Mol Cancer 2: 32–42 14521721 10.1186/1476-4598-2-32 PMC212230

[bib33] Luss H, Klein-Wiele O, Boknik P, Herzig S, Knapp J, Linck B, Muller FU, Scheld HH, Schmid C, Schmitz W, Neumann J (2000) Regional expression of protein phosphatase type 1 and 2A catalytic subunit isoforms in the human heart. J Mol Cell Cardiol 12: 2349–2359 10.1006/jmcc.2000.1265 11113010

[bib34] Merchant MS, Woo CW, Mackall CL, Thiele CJ (2002) Potential use of imatinib in Ewing's sarcoma: evidence for *in vitro* and *in vivo* activities. J Nat Cancer Inst 94: 1673–1679 12441322 10.1093/jnci/94.22.1673

[bib35] Nakatani K, Thompson DA, Barthel A, Sakaue H, Liu W, Weigel RJ, Roth RA (1999) Up-regulation of Akt3 in estrogen receptor-deficient breast cancers and androgen-independent prostate cancer lines. J Biol Chem 274: 21528–21532 10419456 10.1074/jbc.274.31.21528

[bib36] Nishimura N, Furukawa Y, Sutheesophon K, Nakamura M, Kishi K, Okuda K, Sato Y, Kano Y (2003) Suppression of ARG kinase activity by STI571 induces cell cycle arrest through up-regulation of CDK inhibitor p18/INK4c. Oncogene 22: 4074–4082 12821941 10.1038/sj.onc.1206498

[bib37] Ohyashiki K, Ohyashiki JH, Nishimaki J, Toyama K, Ebihara Y, Kato H, Wright WE, Shay JW (1997) Cytological detection of telomerase activity using an *in situ* telomeric repeat amplification protocol assay. Cancer Res 57: 2100–2103 9187102

[bib38] Okada M, Adachi S, Imai T, Watanabe K, Toyokuni SY, Ueno M, Zervos AS, Kroemer G, Nakahata T (2004) A novel mechanism for imatinib mesylate-induced cell death of BCR-ABL-positive human leukemic cells: caspase-independent, necrosis-like programmed cell death mediated by serine protease activity. Blood 103: 2299–2307 14645012 10.1182/blood-2003-05-1605

[bib39] Oshevski S, Le-Bousse-Kerdiles MC, Clay D, Levashova Z, Debili N, Vitral N, Jasmin C, Castagna M (1999) Differential expression of protein kinase C isoform transcripts in human hematopoietic progenitorrs undergoing differentiation. Biochem Biophys Res Commun 263: 603–609 10512725 10.1006/bbrc.1999.1425

[bib40] Pandiella A, Carvajal-Vergara X, Tabera S, Mateo G, Gutierrez N, San Miguel JF (2003) Imatinib mesylate (STI 571) inhibits multiple myeloma cell proliferation and potentiates the effect of common antimyeloma agents. Br J Hematol 123: 858–868 10.1046/j.1365-2141.2003.04706.x 14632777

[bib41] Pang JX, Cheng XY, Xu W, Wu SG (2003) Antisense Sp1 oligodeoxynucleotide decreases telomerase activity by inhibiting hTERT mRNA expression in Jurkat T cells. Acta Pharmacol Sin 24: 91–99 12511235

[bib42] Poremba C, Scheel C, Hero B, Christiansen H, Schaefer KL, Nakayama J, Berthold F, Juergens H, Boecker W, Dockhorn-Dworniczak B (2000) Telomerase activity and telomerase subunits gene expression patterns in neuroblastoma: a molecular and immunohistochemical study establishing prognostic tools for fresh-frozen and paraffin-embedded tissues. J Clin Oncol 18: 2582–2592 10893290 10.1200/JCO.2000.18.13.2582

[bib43] Roussidis A, Mitropoulou T, Theocharis A, Kiamouris C, Papadopoulos S, Kletsas D, Karamanos N (2004) STI571 as a potent inhibitor of growth and invasiveness of human epithelial breast cancer cells. Anticancer Res 24(3a): 1445–1447 15274308

[bib44] Seimiya H, Oh-hara T, Suzuki T, Naasani I, Shimazaki T, Tsuchiya K, Tsuruo T (2002) Telomere shortening and growth inhibition of human cancer cells by novel synthetic telomerase inhibitors MST-312, MST-295, and MST-1991. Mol Cancer Ther 1: 657–665 12479362

[bib45] Shay JW, Zou Y, Hiyama E, Wright WE (2001) Telomerase and cancer. Hum Mol Genet 10: 677–685 11257099 10.1093/hmg/10.7.677

[bib46] Skehan P, Storeng R, Scudiero D, Monks A, McMahon J, Vistica D, Warren JT, Bokesch H, Kenney S, Boyd MR (1990) New colorimetric cytotoxicity assay for anticancer-drug screening. J Natl Cancer Inst 82: 1107–1112 2359136 10.1093/jnci/82.13.1107

[bib47] Suenaga M, Soda H, Oka M, Yamaguchi A, Nakatomi K, Shiozawa K, Kawabata S, Kasai T, Yamada Y, Kamihira S, Tei C, Kohno S (2002) Histone deacetylase inhibitors suppress telmerase reverse transcriptase mRNA expression in prostate cancer cells. Int J Cancer 97: 621–625 11807787 10.1002/ijc.10082

[bib48] Tauchi T, Nakajima A, Sashida G, Shimamoto T, Ohyashiki JH, Abe K, Yamamoto K, Ohyashiki K (2002) Inhibition of human telomerase enhances the effect of the tyrosine kinase inhibitor, imatinib, in BCR-ABL-positive leukemia cells. Clin Cancer Res 8: 3341–3347 12429620

[bib49] Teng L, Chen S, Thomas JF (2002) Stable expression of antisense hTR inhibits *in vitro* pancreatic cancer cell growth. Chin Med J 115: 1196–1200 12215291

[bib50] Teng L, Fahey T (2002) Can inhibition of telomerase increase pancreatic cancer cells susceptibility to chemotherapeutic reagents? Hepatobiliary Pancreat Dis Int 1: 155–160 14607648

[bib51] Vindelov LL, Christensen IJ, Nissen NI (1983) A detergent-trypsin method for the preparation of nuclei for flow cytometric DNA analysis. Cytometry 3: 323–327 6188586 10.1002/cyto.990030503

[bib52] Wang ES, Wu K, Chin AC, Chen-Kiang S, Pongracz K, Gryaznov S, Moore MA (2004) Telomerase inhibition with an oligonucleotide telomerase template antagonist: *in vitro* and *in vivo* studies in multiple myeloma and lymphoma. Blood 103: 258–266 12969977 10.1182/blood-2003-02-0546

[bib53] Wong J, Kusdra L, Collins K (2002) Subnunclear shuttling of human telomerase induced by transformation and DNA damage. Nat Cell Biol 4: 731–736 12198499 10.1038/ncb846

[bib54] Wright WE, Shay JW (2000) Telomere dynamics in cancer progression and prevention: fundamental differences in human and mouse telomere biology. Nat Med 8: 849–851 10.1038/78592 10932210

[bib55] Yi X, Shay JW, Wright WE (2001) Quantitation of telomerase components and hTERT mRNA splicing patterns in immortal human cells. Nucleic Acids Res 29: 4818–4825 11726691 10.1093/nar/29.23.4818 PMC96692

[bib56] Yin T, Wu YL, Sun HP, Sun GL, Du YZ, Wang KK, Zhang J, Chen GQ, Chen SJ, Chen Z (2004) Combined effects of As4S4 and imatinib on chronic myeloid leukemia cells and BCR-ABL oncoprotein. Blood 104: 4219–4225 15339852 10.1182/blood-2004-04-1433

[bib57] Zaffaroni N, Lualdi S, Villa R, Bellarosa D, Cermele C, Felicetti P, Rossi C, Orlandi L, Daidone MG (2002) Inhibition of telomerase activity by a distamycin derivative: effects on cell proliferation and induction of apoptosis in human cancer cells. Eur J Cancer 38: 1792–1801 12175697 10.1016/s0959-8049(02)00139-9

